# The prevalence of mental frailty in ICU survivors and informal caregiver strain: A 1-year retrospective study of the Frisian aftercare cohort

**DOI:** 10.1177/17511437221139547

**Published:** 2022-12-07

**Authors:** Lise F E Beumeler, Carina Bethlehem, Thialda T Hoogstins-Vlagsma, Tim van Zutphen, Hanneke Buter, Gerjan J Navis, E Christiaan Boerma

**Affiliations:** 1 Campus Fryslân, University of Groningen, Leeuwarden, The Netherlands; 2 Department of Intensive Care, Medical Centre Leeuwarden, Leeuwarden, The Netherlands; 3 Department of Medical Psychology, Medical Centre Leeuwarden, Leeuwarden, The Netherlands; 4 Faculty of Medical Sciences, University Medical Centre Groningen, The Netherlands

**Keywords:** Critical illness, mental health, quality of life, frailty

## Abstract

**Background:**

Intensive care unit (ICU) survivors often suffer from long-term mental problems and a reduced health-related quality of life (HRQoL). Symptoms of depression, anxiety, and post-traumatic stress disorder may render patients mentally frail post-ICU, resulting in impaired recovery and an increased informal caregiver burden. The aim of this study was to investigate the prevalence of mental frailty up to 12 months after ICU admission and pinpoint markers for early risk-assessment in clinical practice.

**Methods:**

A retrospective cohort study (2012–2018) in which clinical and post-ICU data of long-stay (⩾48 h) ICU-patients was used. Mental frailty was identified as clinically relevant symptoms of depression, anxiety, or post-traumatic distress disorder at 12 months after discharge.

**Results:**

The prevalence of mental frailty at 12 months post-ICU among the total group of 239 patients was 38%. Mental frailty was defined as clinically relevant symptoms of depression, anxiety, and/or trauma. To achieve this, previously validated cut off values were used for the HADS (HADS-Anxiety ⩾ 8; HADS-Depression ⩾ 8) and TSQ (⩾6), and CSI (⩾7).

**Conclusion:**

A significant proportion of ICU-survivors can be identified as mentally frail, which is associated with impaired HRQoL at baseline and post-ICU, and high caregiver strain. These findings emphasize the need for integrative aftercare programs for both the patient and their informal caregivers.

## Introduction

Mental health-related problems are common in Intensive Care Unit (ICU) survivors and are often associated with limitations in recovery.^1^ Over the past decade, the number of patients surviving critical illness has increased due to advances in intensive care medicine.^2^ Critical illness and admission to the ICU exposes patients to a number of stressors, including but not limited to: invasive medical procedures, delirium, loss of control, unfamiliar surroundings, and the inability to communicate.^3^ A combination of these factors results in a substantial number of ICU-survivors experiencing long-term symptoms of anxiety, depression, and post-traumatic stress disorder.^4,5^ These mental health-related problems can lead to poor health-related quality of life (HRQoL), the hallmark of the Post-Intensive Care Syndrome (PICS).^5^

Impaired mental health and other PICS-related health complaints curtail a patient’s chances of meaningful recovery. Long-term non-recovery in ICU survivors is common^6^ and results in higher healthcare utilization and the inability to participate in society.^7,8^ In addition, long-term impairments pose a high burden on the patient’s informal caregivers, as they often provide informal care. Although the impact of critical illness on relatives and informal caregivers is yet to be fully understood, a large proportion of caregivers experience depressive symptoms, trauma-related problems, and restrictions in daily living over a longer period of time.^9,10^

Although knowledge on mental health-related recovery is expanding, it is still unclear which patients are in need of additional support. Previous studies on recovery in mental health have predominantly studied the presence of isolated symptoms (e.g. anxiety, depression, or trauma-related problems) rather than relevant composite measures such as frailty, and did not use clinically relevant threshold values.^11,12^ Using threshold values for symptoms of depression, anxiety, and trauma may provide an indication of long-term mental frailty. Over the last decade, frailty has been introduced as a predominantly physical phenotype associated with aging and reflecting in higher risk of health problems, care needs, and mortality.^13^ Several frailty indices have been developed to predict morbidity and mortality across the public and clinical health domain. As assessing the frailty phenotype may be troublesome, shorter indices like the clinical frailty scale (CFS) are often used in acute care.^14^ However, these practical tools do not include a comprehensive assessment of mental health and may therefore be less suitable to assess mental frailty and rehabilitation needs in a long-term care setting. Thus, identifying patients with mental frailty can support the search for useful markers of long-term impairments in mental health.

Furthermore, pre-admission mental frailty may affect ICU-survivors in their capability to achieve a meaningful recovery after critical illness. Adding a detailed assessment of psychiatric history, including substance abuse, psychotic disorder, and personality disorders, can help identify specific patients at risk of long-term impairments in mental health. Lastly, including the possibility of an aggravating effect of ICU-related delirium may give essential information regarding the impact of critical illness on long-term mental wellbeing.

To this end, this study aims to investigate the prevalence of mental frailty up to 12 months after ICU admission and pinpoint markers for early risk-assessment in clinical practice. In addition, the presence of psychiatric history or delirium were evaluated as possible predictors for mental frailty. Finally, the impact of mental frailty on patient health-related quality of life and informal caregiver strain was evaluated.

## Methods

### Study design and population

In this retrospective single-center cohort study, all long-stay (⩾48 h) ICU patients visiting the specialized outpatient post-ICU clinic between 2012 and 2018 were included. The study was performed in a tertiary teaching hospital in Leeuwarden, The Netherlands. Following local protocol, long-stay ICU-patients visited the outpatient clinic at 3 months after ICU-discharge. At that time point and 12 months after discharge patients and their informal caregivers completed a predefined standard care set of questionnaires. Data from all patients who completed mental wellbeing assessment were retrieved from electronic patient records. As patient-record data were analyzed anonymously, the ethical committee waived the need for informed consent (Regionale Toetsingscommissie Patiëntgebonden Onderzoek, Leeuwarden, the Netherlands; nWMO-number: nWMO 358).

### Data collection

Information regarding demographics, comorbidities, disease etiology, ICU-morbidity, and post-ICU outcomes were collected from electronic patient records. Psychiatric history was documented as the presence of depression, bipolar disorder, PTSD, anxiety, psychotic illness, personality disorder, or a history of substance abuse based on hospital electronic patient records. Statistics regarding the presence of delirium were obtained from patient records of daily clinical assessment using the Confusion Assessment Method for the ICU (CAM-ICU) and day-to-day status reports.^15^ As part of standard care protocol, several post-ICU outcomes were collected by outpatient clinic professionals at 3 and 12 months after admission.

### Outcome measures HRQoL and mental frailty

In patients, HRQoL was assessed using the Dutch version of the 36-Item Short Form Health Survey (Research and Development-36; RAND-36) (0–100, higher is better).^16^ Mental wellbeing was further assessed using the Hospital Anxiety and Depression Scale (HADS) (0–21 per subscale, higher is worse) and the Trauma Screening Questionnaire (TSQ) (0–10, higher is worse).^17,18^ Coping strategies were assessed using the Coping Inventory for Stressful Situations (CISS-21) (7–35, higher on subscale is more frequent use of task-oriented, emotional, or avoidance coping style).^19^ The survey set for caregivers consisted of the Caregiver Strain Index (CSI) (0–13, higher is worse) and a modified caregiver TSQ (0–10, higher is worse).^20^ Patients were retrospectively allocated to the group with mental frailty or the group with no mental frailty at 12 months. Mental frailty was defined as clinically relevant symptoms of depression, anxiety, and/or trauma. To achieve this, previously validated cut off values were used for the HADS (HADS-Anxiety ⩾ 8; HADS-Depression ⩾ 8) and TSQ (⩾6), and CSI (⩾7).

### Statistical analysis

Patient and caregiver data were stored in a coded data file in a dedicated research directory. Demographics, comorbidities, disease etiology, ICU-morbidity, and post-ICU outcomes are presented for cases with mental frailty and patients without. All data were visually inspected for normality and underwent normality testing using a Shapiro-Wilk approach. As the majority of data were found to be non-normally distributed, results are displayed as median and [interquartile range, IQR] or percentages in case of categorical data. Within-group analyses were conducted using Wilcoxon signed rank tests for continuous data and Related-Samples McNemar Change tests for dichotomous data. Between-group assessment was performed using Mann-Whitney U tests for continuous data or a Chi-square tests when comparing group proportions.

Odds ratios (OR) for mental frailty at 12 months post-ICU were calculated using binary logistic regression analysis after assessing for collinearity. With this analysis, the impact of clinically relevant characteristics at baseline (delirium in ICU and psychiatric history) and 3 months mental wellbeing scores (trauma, anxiety, depression, and coping scores) on the odds for mental frailty was assessed. For trauma, anxiety, and depression scores, binary variables were used in the model to indicate if patients reached cut-off values at 3 months. A second model was analyzed using absolute trauma, anxiety, and depression scores at 3 months. The analyses were adjusted for sex, age, and severity of illness (APACHE III). A 2-sided *p*-value of 0.05 was considered statistically significant for all results. Analyses were performed in IBM SPSS Statistics for Windows, version 27.0 (IBM, NY, USA). Figures were designed using GraphPad Prism, version 9 (GraphPad Software, CA, USA). A size-proportional Venn diagram was made using the online BioVenn tool.^21^

## Results

### Study inclusion and population

**Figure 1.**
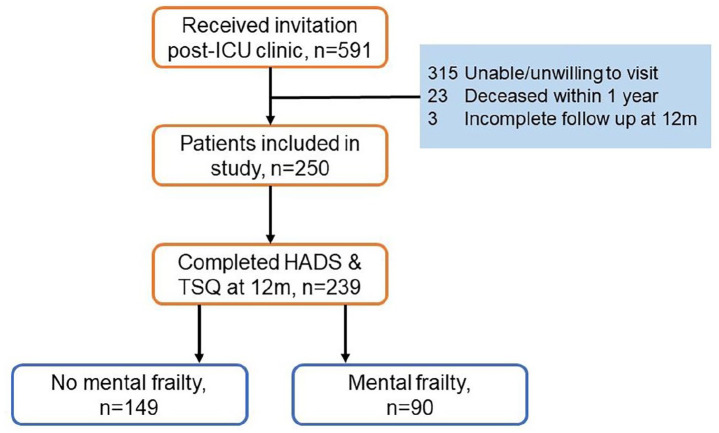
Flowchart of study inclusion.

Of the 250 patients that were eligible for the study, a total of 239 patients completed mental health assessment at 12 months after ICU discharge (Figure 1). Based on this 12-month assessment, 90 patients (38%) were allocated to the mental frailty group (Table 1). One hundred forty-nine patients were assigned to the group with no mental frailty. There was no difference between groups in sex, age, and severity of illness. Regarding important comorbidities, a difference was found in the total amount of patients with any psychiatric history and specifically in the incidence of pre-existing post-traumatic stress disorder (PTSD). Finally, ICU-characteristics were found to be identical in the two groups.

**Table 1.** Baseline and ICU-characteristics after group allocation at 12 months post-ICU.

	No mental frailty, *n* = 149 (62%)	Mental frailty, *n* = 90 (38%)	*p*-value
Demographic factors			
Female, n (%)	57 (38)	35 (39)	0.992
Age	67 [57–73]	67 [57–74]	0.785
BMI	26.5 [24.3–29.4]	26.4 [23.5–30.1]	0.396
APACHE III	81 [60–101]	73 [56–91]	0.102
Comorbidities			
Medical comorbidity*, n (%)	64 (43)	48 (53)	0.119
Psychiatric history, n (%)	21 (14)	22 (24)	**0.044**
Depression	9 (6)	12 (13)	0.054
Bipolar dis.	0	1 (1)	0.197
PTSD	0	4 (4)	**0.009**
Anxiety dis.	2 (1)	4 (4)	0.137
Psychotic dis.	0	0	NA
Personality dis.	3 (2)	2 (2)	0.913
Substance abuse	10 (7)	3 (3)	0.265
>1 psychiatric disorder, n (%)	3 (2)	5 (6)	0.157
Etiology			
Admission, n (%)			
Medical	70 (47)	51 (57)	0.343
Elective surgical	29 (19)	15 (17)	
Acute surgical	50 (34)	24 (27)	
Sepsis, n (%)	49 (33)	32 (36)	0.673
CPR, n (%)	30 (20)	15 (17)	0.506
ICU morbidity			
LOS ICU	10 [6–20]	12 [6–23]	0.243
Mech. vent (days)	6 [3–12]	6 [3–17]	0.650
Delirium, n (%)	66 (44)	42 (47)	0.721

APACHE: acute physiology and chronic health evaluation; CPR: cardiopulmonary resuscitation; LOS ICU: length of stay intensive care unit. Bold values indicate significant p-value.

* Medical comorbidity: history of malignancy, diabetes, chronic obstructive pulmonary disease, stroke, and/or renal disease

### Mental wellbeing of patients and caregivers at 3 and 12 months after discharge

**Figure 2.**
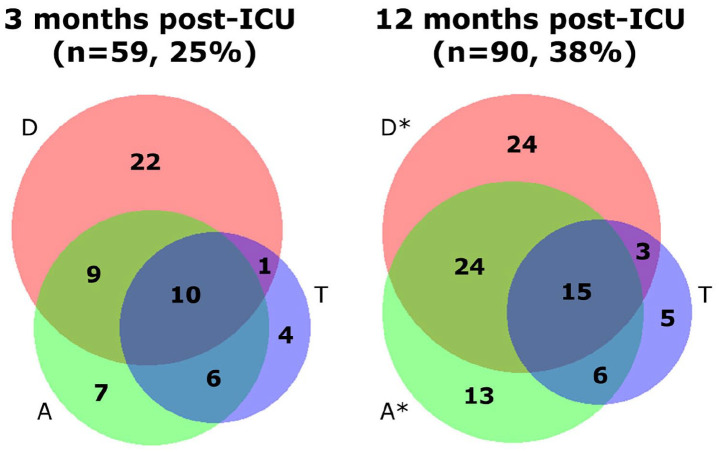
Size-proportional Venn diagram of the presence of (a combination of) above threshold depressive symptoms (D, red), anxiety (A, green), or trauma (T, blue) in all patients at 3 and 12 months post-ICU in absolute numbers with significant increases indicated as *p ⩽ 0.05.

The percentage of patients experiencing mental frailty, previously described as at least one clinically relevant psychiatric symptom, increased over time (25% vs 38%, *p* = 0.010, Figure 2). The increase is most prominently present in isolated or combined anxiety or depressive symptoms (13%–24%, *p* = 0.017, 17%–27%, *p* = 0.045). If patients experienced a combination of psychiatric symptoms, it was most often depression and anxiety at both time points (4% and 10%, respectively). A marginal proportion of patients with mental frailty experienced a combination of depression, anxiety, and trauma-related issues.

**Figure 3.**
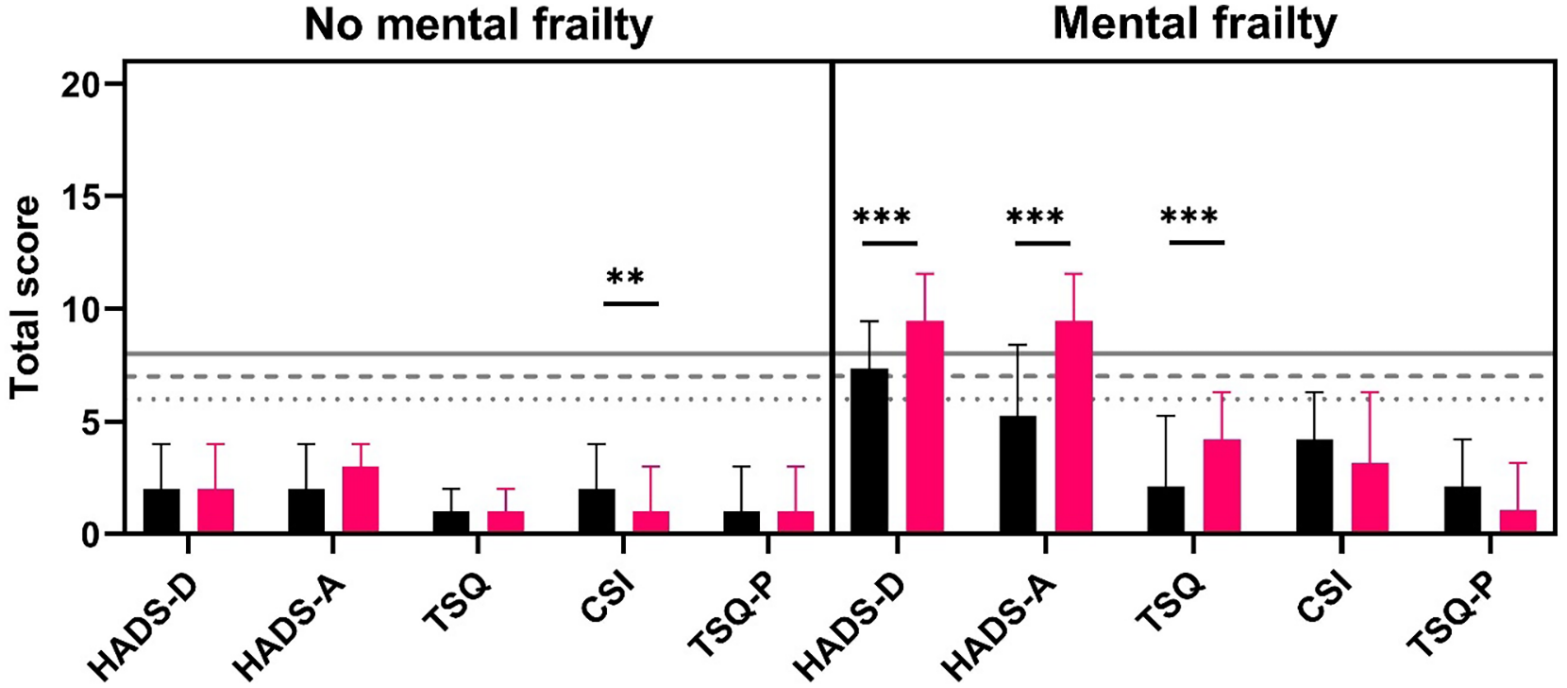
Absolute scores in depression (HADS-D), anxiety (HADS-A), and trauma (TSQ) (median, 95% CI) for patients with or without mental frailty combined with caregiver strain (CSI) and caregiver trauma (TSQ-P) at three (black) and 12 (red) months. Dotted line, threshold TSQ; interrupted line, threshold CSI; continuous line, threshold HADS. ^**^p < 0.01 ^***^p < 0.001.

Univariate analyses of patient mental health scores and caregiver strain indices singled out several differences between groups (Figure 3). At both time points, patients with mental frailty scored higher for anxiety, depression, and trauma symptoms. Caregivers of patients with mental frailty experienced higher strain and had more trauma-related complaints at 3 and 12 months. At 3 months, 20% of caregivers of patients with mental frailty experienced high levels of strain compared to 11% in the non-frail group (*p* = 0.042). This difference in caregiver strain was similar at 12 months post-ICU (22% vs 6%, *p* < 0.001). Moreover, absolute CSI scores decreased over time in the non-frail group. In addition, caregivers of patients with worse mental health outcomes more often experienced above threshold trauma symptoms at 12 months (17% vs 3%, *p* < 0.001) (Supplemental File 1).

Patients with mental frailty more often reported the use of avoidance (at 12 months) and emotional coping strategies (at 3 and 12 months) (Table 2). The use of emotional coping strategies increased over time in the mental frailty group.

**Table 2.** Patient coping with stressful situations at 3 and 12 months after discharge. *p*-values in far right column indicate between-group significance.

Coping with stressful situations		No mental frailty	Mental frailty	*p*-value
CISS-21, avoidance (7–35)	3 months	17 [12–22]	19 [13–22]	0.585
	12 months	16 [11–22]	19 [13–22]	**0.033**
CISS-21, task (7–35)	3 months	18 [13–23]	20 [16–24]	0.061
	12 months	20 [13–25]	20 [17–23]	0.838
CISS-21, emotional (7–35)	3 months	14 [9–18]	18 [13–21]	<**0.001**
	12 months	13 [8–18]	20 [14–24]	<**0.001**

Bold values indicate significant p-value.

**Figure 4.**
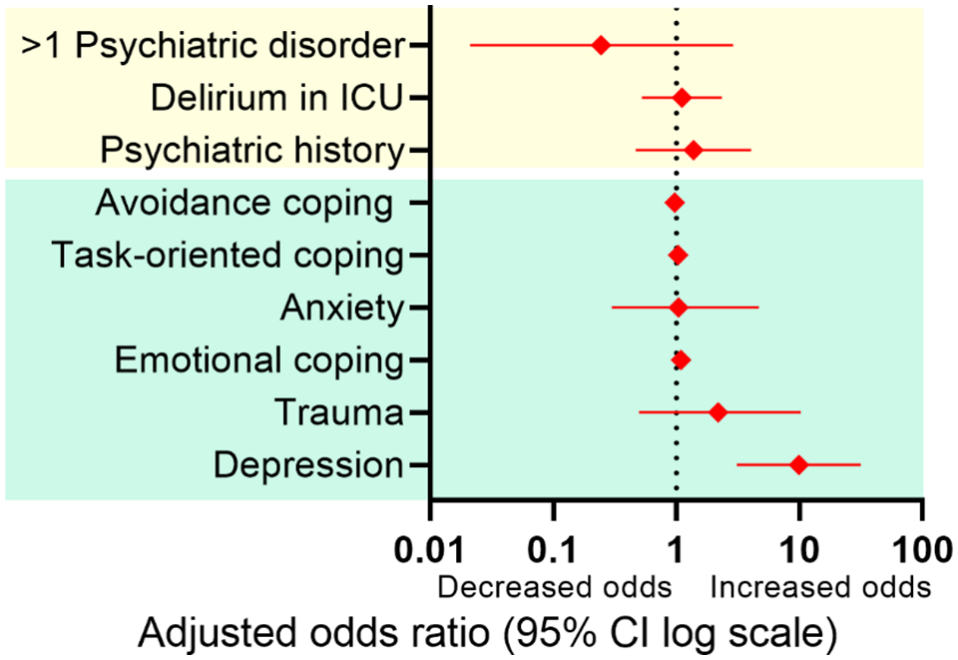
Adjusted odds ratios for mental frailty 1 year post-ICU for baseline, ICU (in yellow), and 3 months post-ICU characteristics (in green), corrected for age, sex, and severity of illness.

Using multivariate analysis, depression score of ⩾8 at 3 months was identified as a statistically significant marker for an almost 10-fold increase in odds of mental frailty at 12 months (OR: 9.869 [95% CI 3.100–31.417]; Nagelkerke *R*^2^ = 0.320, Figure 4). When using absolute depression, anxiety, and trauma scores at 3 months for a second model, an increase of one point on absolute depression score at 3 months resulted in an almost 1.4 increase in odds of mental frailty at 12 months after ICU-discharge (OR: 1.361 [95% CI 1.168–1.586]; Nagelkerke *R*^2^ = 0.386).

### Impact of frailty on mental health-related quality of life

**Figure 5.**
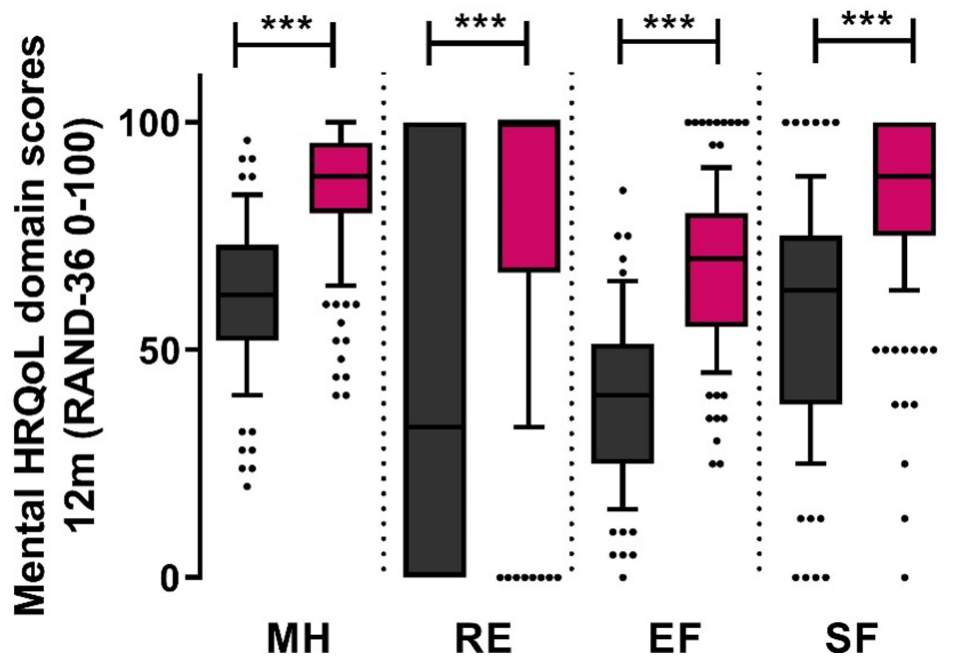
Mental HRQoL at 12 months after discharge comparing patients with mental frailty (black) after 1 year to patients with no mental frailty (red). MH: mental health; RE: problems in role functioning due to emotional problems; EF: energy/fatigue; SF: social functioning. ^***^p < 0.001.

Lastly, between-group univariate analyses indicated that at 3 and 12 months post-ICU patients with mental frailty scored significantly lower for mental health-related domain scores of the RAND-36. Patients experiencing relevant symptoms of anxiety, depression, or trauma at 3 months, scored lower when it comes to mental health (MH; 68 [48−76] vs 88 [76−94]), impaired role functioning due to emotional problems (RE; 33 [0–67] vs 100 [67–100]), energy/fatigue (EF; 40 [30−55] vs 65 [55−75]), and social functioning (SF; 50 [38–75] vs 88 [63–100]) (*p* < 0.001). Similar patterns were observed at 12 months where frail patients scored significantly lower with regards to mental HRQoL (Figure 5).

## Discussion

This study demonstrated that more than a third of ICU survivors are mentally frail 12 months after ICU-admission. The number of patients with mental frailty increased between 3 and 12 months, illustrating the progressive nature of these serious mental health problems. Symptoms of depression were the most prominent mental health complaints throughout the year, with anxiety-related issues as a runner-up. Mental frailty had a detrimental impact on mental HRQoL and caregiver burden, suggesting a wide spread influence of frailty on patients’ daily lives. Although psychiatric history and delirium did not significantly predict long-term mental frailty, higher depression scores (⩾8 on HADS-D) at 3 months resulted in a tenfold increase in odds and may be an appropriate marker for early detection of patients at risk.

Mental frailty is a new and relatively unexplored aspect of long-term vulnerability in patients and healthy individuals. Despite the generally physical focus within the concept of frailty, an increasing level of attention has been attributed to the impact of mental and cognitive wellbeing on health and susceptibility to disease.^22,23^ In this study, using a mental frailty definition based on threshold levels of depression, anxiety, and trauma, 38% of patients were identified as “frail” after 12 months. A previous study in the post-acute setting showed that anxiety, depression, and trauma-related symptoms were common at 1 year after discharge.^4^ Similar to findings in the current study, there was a large overlap between mental health problems (63% experienced a combination of anxiety, depression, or trauma-related symptoms), supporting the use of a mental frailty framework. A second study among 629 patients found that close to a third was suffering from symptoms of anxiety, followed by PTSD and depression at 3 months after discharge.^5^ Despite a rather large variation in the percentage of patients with mental health problems and heterogeneity in distribution of symptoms, the current study confirms that mental frailty is common in ICU survivors.

The general assumption is that a psychiatric history before admission and presence of delirium during admission make patients more vulnerable for long-term mental morbidity.^24^ However, current data suggest that these factors have a limited impact on the odds for mental frailty when including mental health parameters to the prediction model. Previous studies confirmed the lack of association between psychiatric history, delirium, and long term mental health.^4,25^ Our model identified higher depression scores at 3 months as a strong predictor for long-term mental frailty. These findings suggest that using psychological screening tools for depression, among others, may be an effective screening tool for mental frailty in the post-critical illness rehabilitation setting. In addition, including sub-clinical depression scores in post-ICU assessments of recovery may be necessary to properly identify patients in need of after care as symptoms in patients with frailty may worsen over the year.

Long-term mental frailty has far-reaching consequences for the patient and their informal caregivers. In this study, patients with mental frailty scored significantly lower on several domains of HRQoL, namely mental health, problems in role functioning due to emotional problems, energy/fatigue, and social functioning. Specifically in the domains of role functioning and energy/fatigue patients with mental frailty did not match age-adjusted reference values.^16^ In addition, patients that were experiencing mental frailty at 12 months scored higher on emotional coping at both time points, which may be an interesting finding to explore further in future research. The difference in coping may also be a contributing factor of our finding that mental frailty was correlated with higher caregiver burden and caregiver trauma symptoms. At both 3 and 12 months, a fifth of caregivers indicated unhealthy levels of strain. Although the subject of caregiver burden and PICS-Family (PICS-F) are still underrepresented in medical outcome research, a study of 94 relatives of ICU survivors found similar results.^9^ Additional studies found a correlation between impaired HRQoL of the patient and caregiver burden.^26,27^ These findings may be self-evident, aside from the fact that caregiver strain remained high even after patient health status improved. Such discrepancy indicates that it’s essential to include caregivers of patients in ICU aftercare practices.

Although this study comprises valuable data regarding mental frailty post-ICU, there are some limitations to consider. To our knowledge, this is the first time a mental frailty construct is built from the presence of above threshold symptoms of depression, anxiety, and trauma. Nevertheless, the scales used for identification of mental frailty have not been validated for this purpose. To illustrate, even though the TSQ has been validated and used frequently to evaluate post-traumatic stress disorder-related symptoms after critical illness in patients and informal caregivers, it may be less suitable to assess complex trauma or the long-term impact of the often traumatic ICU admission.^9,28^ For research purposes this construct provides valuable information of a patient’s mental vulnerability during ICU-recovery. However, in clinical practice frailty encompasses not only mental health, and both physical and cognitive health should be taken into account when assembling a risk profile. In addition, depression scores at 3 months were included in our prediction model while depression scores at 12 months were used in the outcome measure. Although both variables passed the collinearity test, this could have an impact on the prediction model. Furthermore, despite including extensive information on psychiatric history, this study did not use data on medication use during ICU-stay, sedation techniques, and other factors that may have affected mental recovery.

In conclusion, there is a large proportion of ICU-survivors with increasing mental frailty in the first 12 months after critical illness. Psychiatric history at admission and delirium in ICU did not increase the odds for unfavorable outcome. However, depression scores at 3 months may be used as a marker for long-term mental frailty in clinical practice. Future research is needed to investigate the individual trajectories of mental health-related symptoms to uncover the cause of increased depression and anxiety-related symptoms throughout the first year of ICU recovery. Mixed-method studies, combining qualitative and quantitative outcomes, could provide valuable insights on the nature and extent of long-term mental health-related problems in ICU survivors. Mental frailty results in HRQoL impairments and higher caregiver burden, which emphasizes the need for integrative aftercare programs for both the patient and their informal caregivers.

## Supplemental Material

sj-docx-1-inc-10.1177_17511437221139547 – Supplemental material for The prevalence of mental frailty in ICU survivors and informal caregiver strain: A 1-year retrospective study of the Frisian aftercare cohort Click here for additional data file. Supplemental material, sj-docx-1-inc-10.1177_17511437221139547 for The prevalence of mental frailty in ICU survivors and informal caregiver strain: A 1-year retrospective study of the Frisian aftercare cohort by Lise F E Beumeler, Carina Bethlehem, Thialda T Hoogstins-Vlagsma, Tim van Zutphen, Hanneke Buter, Gerjan J Navis and E Christiaan Boerma in Journal of the Intensive Care Society
