# Supplementary material for: The prevalence of mental frailty in ICU survivors and informal caregiver strain: A 1-year retrospective study of the Frisian aftercare cohort
Source: J Intensive Care Soc. 2022 Dec 7;24(4):356–63. doi: 10.1177/17511437221139547 (PMC10572478; doi:10.1177/17511437221139547)
Supplement: sj-docx-1-inc-10.1177_17511437221139547 – Supplemental material for The prevalence of mental frailty in ICU survivors and informal caregiver strain: A 1-year retrospective study of the Frisian aftercare cohort [file sj-docx-1-inc-10.1177_17511437221139547.docx]

Supplemental table 1: Mental health and caregiver strain of informal caregivers and patients. Significant differences in bold.

| **Patient mental health** | | No mental frailty | Mental frailty | *p*-value |
| --- | --- | --- | --- | --- |
| HADS, anxiety  (0-21) | 3 months | 2 [1-4] | 5 [3-8] | **<0.001** |
|  | 12 months | 2 [1-4] | 9 [7-11] | **<0.001** |
| HADS, depression (0-21) | 3 months | 2 [0-4] | 7 [4-9] | **<0.001** |
|  | 12 months | 3 [1-4] | 9 [6-11] | **<0.001** |
| TSQ  (0-10) | 3 months | 1 [0-2] | 2 [1-5] | **<0.001** |
|  | 12 months | 1 [0-2] | 4 [2-6] | **<0.001** |
| **Caregiver strain** |  |  |  |  |
| CSI (0-13) | 3 months | 2 [0-4] | 4 [1-6] | **0.005** |
|  | 12 months | 1 [0-3] | 3 [1-6] | **<0.001** |
| CSI ≥ 7 | 3 months | 16 (11) | 18 (20) | **0.042** |
| n (%) | 12 months | 9 (6) | 20 (22) | **<0.001** |
| Caregiver TSQ | 3 months | 1 [0-3] | 2 [0-4] | **0.038** |
| (0-10) | 12 months | 0 [0-2] | 1 [0-3] | **<0.001** |
| Caregiver TSQ ≥ 6 | 3 months | 9 (6) | 16 (18) | **0.004** |
| n (%) | 12 months | 4 (3) | 15 (17) | **<0.001** |
